# Family resilience of families with parental cancer and minor children: a qualitative analysis

**DOI:** 10.3389/fpsyg.2023.1251049

**Published:** 2024-01-19

**Authors:** Christian Heuser, Juliane Nora Schneider, Lina Heier, Nicole Ernstmann, Hannah Nakata, Andrea Petermann-Meyer, Rebecca Bremen, André Karger, Andrea Icks, Tim H. Brümmendorf, Franziska Geiser

**Affiliations:** ^1^Chair of Health Services Research, Institute of Medical Sociology, Health Services Research, and Rehabilitation Science, Faculty of Medicine and University Hospital Cologne, University of Cologne, Cologne, Germany; ^2^Center for Health Communication and Health Services Research, Clinic and Policlinic of Psychosomatic Medicine and Psychotherapy, University Hospital Bonn, Bonn, Germany; ^3^Center for Integrated Oncology Aachen Bonn Cologne Düsseldorf (CIO ABCD), Aachen, Germany; ^4^Clinic and Policlinic of Psychosomatic Medicine and Psychotherapy, University Hospital Bonn, University of Bonn, Bonn, Germany; ^5^Department of Clinical Pharmacy and Toxicology, Maastricht University Medical Center, Maastricht, Netherlands; ^6^CARIM School for Cardiovascular Disease, Maastricht University, Maastricht, Netherlands; ^7^Department of Hematology, Oncology, Hemostaseology and Stem Cell Transplantation, Faculty of Medicine, RWTH Aachen University, Aachen, Germany; ^8^Clinical Institute for Psychosomatic Medicine and Psychotherapy, Medical Faculty and University Hospital Düsseldorf, Heinrich-Heine University Düsseldorf, Düsseldorf, Germany; ^9^Institute for Health Services Research and Health Economics, Faculty of Medicine, Heinrich-Heine University of Düsseldorf, Düsseldorf, Germany; ^10^Institute for Health Services Research and Health Economics, German Diabetes Center, Düsseldorf, Germany

**Keywords:** parental cancer, minor children, resilience, family intervention, family-SCOUT, oncology, qualitative research and analysis, health services research

## Abstract

**Introduction:**

Estimated 50,000 minor children in Germany experience a newly diagnosed cancer in one of their parents every year. Family resilience has proven to be an important concept against life crises. However, little research exists regarding family resilience in the context of parental cancer with minor children. Based on the “Family Resilience Framework,” the aim of the study is to investigate the processes of family resilience of affected families. In addition, we explore which combinations of promoting family resilience processes can be characterized.

**Methods:**

As part of the mixed-method quasi-experimental interventional study “F-SCOUT,” a qualitative content analysis was used to analyze the documentation of the “Family-Scouts” (a fixed contact person who advises, accompanies, and supports the families). Documentation was performed by families’ study inclusion (T0), after 3 months (T1) and 9 months (T2) concerning current family situation, organization of everyday life, emotional coping, open communication within the family, and planned tasks.

**Results:**

The *N* = 73 families had between one and six children. In 58 (79%) families, the mother had cancer. In the course of the analysis, a category system with 10 main categories and 36 subcategories emerged. Family resilience processes were described to different extents. Combinations of categories promoting family resilience were characterized by the use of social resources, flexibility, economic resources, and open communication.

**Discussion:**

The findings are consistent with existing assumptions about family resilience in terms of the importance of social resources, family cohesion, mutual support, flexibility, open communication, and psychological well-being. In contrast to the findings of previous research, spirituality, and collaborative problem-solving indicate less centrality here. In turn, the findings on economic resources and information-seeking provide a valuable addition to the family resilience literature in the context of parental cancer with minor children.

**Clinical trial registration:**

ClinicalTrials.gov, identifier NCT04186923.

## Introduction

According to current estimates, 498,000 people receive a cancer diagnosis in Germany every year (Robert Koch-Institute, 2022a). Many of the patients do not face the diagnosis alone. A 2010 survey from the United States showed that 18% of cancer patients had minor children at initial diagnosis (Weaver et al., 2010). In Germany, 50,000 minor children experience a newly diagnosed parental cancer yearly, according to current estimates (Robert Koch-Institute, 2022b). Parental cancer affects not only the individual but the entire family (Veach and Nicholas, 1998). In addition to the negative impact of the diagnosis on the quality of life (Annunziata et al., 2013; Bellardita et al., 2013), well-being (Heinemann and Simeit, 2017), and mental health (Romer, 2007; Hinz et al., 2010; Nakaya et al., 2010; Götze et al., 2012) of individual family members, the disease affects both family functionality (Kühne et al., 2013) and relationships among family members (Drabe et al., 2013; DellaRipa et al., 2015). Furthermore, parental cancer can be associated with intense stress in children (Faccio et al., 2018). Compared with children in the general population, children of parents with a serious physical illness are about twice as likely to develop psychological symptoms (Romer, 2007). Among the most common of these are anxiety and depressive phenomena, and psychosomatic complaints. In addition, parental cancer can be a potentially traumatic experience for a growing child (Howell et al., 2016).

To support families with parental cancer in developing an adaptive approach to the disease, research has shifted from a deficit to a resource perspective (Buchbinder et al., 2009). The focus is often on resilience as a significant protective factor against stressful life events and crises (Seiler and Jenewein, 2019). The term resilience then primarily refers to “psychological resilience” (Bengel and Lyssenko, 2012; Kurz et al., 2014), which enables individuals to maintain or restore their mental health in the context of adverse circumstances and stresses (Kalisch, 2017). In oncology, research on resilience has focused primarily on individuals, finding positive associations between resilience and general well-being (Bajjani-Gebara et al., 2019) as well as higher quality of life and better mental health (Kurz et al., 2014; Molina et al., 2014; Li et al., 2016) of cancer patients. The concept of “maintaining mental health” in resilience research is often reduced to not developing psychiatric symptoms after confrontation with adversities (Hiebel et al., 2021). A developmental definition describes resilience as a positive adaptation process with the result of maintained or regained psychological and social functioning (Luthar et al., 2000). Masten adds that studying resilience should include a systemic approach, taking into account the networks of relationships and community support (Masten, 2001). Another definition by Yehuda views resilience as a reintegration of self that includes a conscious effort to move forward in an insightful integrated positive manner as a result of lessons learned from an adverse experience (Southwick et al., 2014). When transferring these concepts to family systems, a resilient family may well experience psychological distress, but will find ways to adapt to the new situation, activate networks, and maintain a positive outlook which allows the family members to move on as a family as well as in their individual lives.

Studies examining resilience in a family with parental cancer are scarce (Buchbinder et al., 2009; van Schoors et al., 2015). Family resilience is often operationalized as the healthy and successful functioning of a family following significant stress (Walsh, 2003; van Schoors et al., 2015). Within the family system, this includes dynamic, interactive, and familial processes (Faccio et al., 2018) that promote successful coping (Burgette, 2020) and adaptation to stresses and crises (Luthar et al., 2000; van Schoors et al., 2015). The processes of family resilience enable the family system to recover from crises, buffer stress, reduce the risk of dysfunction, and support optimal adjustment (Walsh, 2003).

In this research, the theoretical basis of understanding family resilience is the “Family Resilience Framework” (Walsh, 2003) which captures effective key family processes in crisis situations. According to Walsh, dynamic and effective family processes and associated interactions are central in the collective coping with a stressful life event like parental cancer (Walsh, 2003). The framework identifies three areas: (1) Family belief systems containing subcategories “Make meaning of adversity,” “Positive outlook” and “transcendence and spirituality”; (2) Family organizational patterns containing “Flexibility,” “Connectedness” and “Social and economic resources”; (3) Family communication / Problem solving containing “Clarity,” “Open emotional expression” and “Collaborative problem-solving.”

It has been emphasized by researchers in the past that families sometimes show remarkable resilience in dealing with the cancer of a family member (Gerhardt et al., 2007; Jantien Vrijmoet-Wiersma et al., 2008; Buchbinder et al., 2009; Chen et al., 2018). However, there is a lack of concrete research on the underlying processes of family resilience in families with parental cancer and minor children. Therefore, the aim of this study is to exploratively examine and describe which processes of family resilience take place in families with parental cancer and minor children. Of particular interest in this context are factors that help families to cope with the crisis. Hence, it will be investigated which, according to Walsh’s model, resilience-promoting (combinations of) family resilience categories can be observed.

## Methods

The methods section is based on the “Consolidated criteria for reporting qualitative research (COREQ),” a 32-item checklist for interviews and focus groups (Tong et al., 2007), in order to support the validity and reliability of the qualitative content analysis.

### Study design

This study is part of the larger mixed-method quasi-experimental interventional study “Family-SCOUT” (Comprehensive support for families with parental cancer – Family-SCOUT) supported by the German Innovation Fund and conducted at the Cancer Centers of the University Hospitals of Aachen, Bonn, and Düsseldorf between July 2018 and June 2022. The study followed a convergent-parallel mixed-methods design (O'Cathain et al., 2010). The intervention consisted of active outreaching, cross-sectional and cross-phase support for families with parental cancer and minor children by the so-called “Family-Scouts,” who are a fixed contact person who advises, accompanies, and supports the families concerning organizational, emotional, and communicative purposes and facilitates access to existing support services (Dohmen et al., 2021). The primary study aim was to examine the effectiveness of the intervention. Further information on the study design can be found elsewhere (Dohmen et al., 2021).

### Sample and data collection

Families with parental cancer and minor children were consecutively recruited in the Centers for Integrated Oncology in the German University Hospitals Aachen, Bonn, and Düsseldorf. Inclusion criteria were a confirmed ICD-10 diagnosis of cancer in one parent, custody of at least one minor child (and/or one minor living in the household), age > 18 years, sufficient German language skills, membership of the cancer-diagnosed parent in German statutory health insurance, and written informed consent from at least the cancer diagnosed parent (or the existence of a written procuration for the healthy parent).

Here, data from the Center for Integrated Oncology Bonn is used. In the intervention group, the Family-Scouts accompanied the families throughout the course of the disease (or even after the death of the parental cancer patients). The Family-Scouts collected the data by completing standardized documentation forms (see Supplementary materials) at three time points which corresponded with the quantitative measurement time points: at study inclusion of the whole family (T0 baseline), 3 months (T1) and 9 months (T2) after study inclusion. The standardized documentation forms were completed after an appointment with the families at the appropriate time T0-T2. The duration of the accompaniment by the Family-Scout (=intervention) was individually adapted to the needs of the families. In this study, the targeted period was at least nine months. Termination of contact with the Family-Scouts or withdrawal of consent to the study resulted in a reduced number of completed documentation forms in some families.

### Measure

The Family-Scouts documented the organizational, emotional, and communicative situation in the families of the intervention group on standardized documentation forms at the three time points T0-T2 (see Supplementary materials) with the help of open text formats or check boxes, e.g., concerning the situation of the families with regard to the disease, family characteristics, household management, care situation of the children, organizational factors, emotional coping with the disease, assessment of the (open) communication in the families, as well as work assignments and action plans to be derived (Dohmen et al., 2021).

### Data analysis

VERBI MAXQDA 2020 software was used for analysis. The Family-Scouts documentation sheets were checked for completeness and matched by identification numbers over the three time points for each family. A structuring qualitative content analysis according to Kuckartz was conducted including seven phases (Kuckartz and Rädiker, 2020, 2022): (1) initiating text screening, (2) derivation of deductive main categories, here from the “Family Resilience Framework” (Walsh, 2003), (3) first coding of the whole data with the main categories, (4) coded segments of the main categories were compiled into lists and discussed, (5) coded segments of the main category were deductively [Family Resilience Framework (Walsh, 2003)] and inductively (by means of the data) systematized and differentiated into subcategories leading to a differentiated category system, (6) second coding of the whole data with the differentiated category system, and (7) in-depth analysis of main and subcategories, here concerning which processes of family resilience take place in families documented by the Family-Scouts were observable. Subsequently, it was examined which (combinations of) main and subcategories were coded that theoretically are resilience-promoting (according to Walsh’s model), sometimes with the help of complex code configurations to investigate multidimensional relationships between categories. To increase the validity of the qualitative content analysis, data were analyzed by two researchers (JNS, CH) independently with different professional backgrounds (psychology and sociology) and both with 3–8 years of experience in qualitative research and coding. Regular meetings were held during the coding process to discuss coding segments and category systems. Interrater reliability was based on Cohen’s Kappa calculation for a random sample of 10 families. The agreement was substantial (*κ* = 0.69).

IBM SPSS version 29 was used to analyze the sociodemographic data (age, number of children, age of parents, sex of the parental cancer patient) of the sample descriptively.

## Results

### Sample description

From the *N* = 78 families in the intervention group in Bonn, data for *N* = 73 families were available for analysis. Data on age and number of children were available for *N* = 70 families. The families had one to six children (*M* = 1.97, SD = 1.12) with an average age of *M* = 9.44 years (SD = 5.46, range 0–27). Sociodemographic data were available for *N* = 70 parental cancer patients and *N* = 57 cancer-free parents. Parental cancer patients were *M* = 46 years old (SD = 6.53, range 32–64) and cancer-free parents were *M* = 48 years old (SD = 7.43, range 31–62). In 58 (79%) families, the mother had cancer, in 16 (22%) the father, and in one (1%) family both parents. In 16 (22%) families the parent with cancer died during the approximately 9 months of intervention. In 11 families (15%), the support by the Family-Scouts had a duration shorter than 9 months, because Family-Scouts (in accordance with the families) saw no further need for support. 21 (29%) families broke off contact with the Family-Scouts over the course of the 9 months by no longer responding to their attempts to contact them. For 51 (70%) families three filled out documentation forms (T0, T1, T2) were available. All available and also incomplete documentation forms were analyzed.

### Qualitative analysis: family resilience in families with parental cancer and minor children

In the course of the analysis, a category system with 10 main categories and 36 subcategories emerged. For each main category 0 to 8 subcategories were differentiated. The number of coded segments of the main categories ranged from 4 (Transcendence and Spirituality) to 208 (Social Resources). The complete coding tree is shown in Table 1. The Family Resilience Framework combines economic and social resources into one category. However, based on the data material and the inductive coding it was decided to separate this category into two.

(1) Family belief systems – Make meaning of adversity: The parental cancer disease was not described as meaningful *per se* to the Family-Scouts. Rather, the illness or the death of a parent was perceived as manageable or normalized.
(2) Family belief systems – Positive outlook: Confidence, hope, courage, and perseverance were demonstrated by many families. One pattern contained the acceptance of the disease and associated physical limitations, while the other pattern consisted in managing ambivalence through phases of repression or denial. For most of the families, psychological and emotional stress, as well as fear, sadness, helplessness, excessive demands, and despair, were evident in the documentation of the Family-Scouts.
(3) Family belief systems – Transcendence and spirituality: In the documentation forms of the Family-Scouts a few text passages associated with transcendence and spirituality were found. No subcategories were developed because there were only four coded segments. Spirituality and transcendence appeared in the form of mostly organizational help from other members of the religious community or social environment.
(4) Family organization patterns – Flexibility: The analysis showed that helpful aspects of flexibility, such as restructuring and adapting to the new situation as well as obtaining information about the disease, were often (inductively) found in the documentation. However, a few families found it difficult to adapt to the new living circumstances or were hardly or only moderately informed.
(5) Family organization patterns – Connectedness: Family connectedness varied greatly among the families. In some families, strong cohesion and/or mutual support was evident for the Family-Scouts. In others, however, there was little cohesion and no or a lack of mutual support. Some family members withdrew from joint family life. In addition, in some families, the parents were separated, and/or there were problems or conflicts in contact with each other.
(6) Family organization patterns – Social resources: There were numerous social resources. Family members and friends helped in the household, took the children to school, or looked after them when the parents did not have time for various reasons (e.g., chemotherapy, work). The professional support system, such as psycho-oncologists, psychotherapists, or the Family-Scouts, was also frequently used. However, there were also families that did not have or use social support.
(7) Family organization patterns – Economic resources: Some families were in sufficient financial positions with parents in steady employment or reporting financial reserves. Other families received financial support from the state or relatives. Unemployment, no financial reserves, or debt was also reported to the Family-Scouts and problematized.
(8) Family communication and problem-solving – Clarity: The family systems differed greatly in how openly they communicated about parental cancer or about other topics in general. The Family-Scouts reported a range from very open communication to intentional concealment.
(9) Family communication and problem-solving – Open emotional expression: The Family-Scouts documented that some families shared their emotions and dealt with each other empathically. In others, however, emotional closeness, control of emotions, or lack of emotional exchange was observed.
(10) Family communication and problem-solving – Collaborative problem-solving: Joint problem-solving was characterized by the joint development of solutions or the collective work on opportunities to solve a problem (e.g., by actively asking for help). In contrast, in families where problems concerning parental couple relationships arose, (couple) conflicts were not managed together and decisions were not made together.

**Table 1:** Differentiated category system of family resilience of families with parental cancer and minor children (*N* = 73 families).

Main and subcategories	Number of codes segments	Examples for documentation from the Family-Scouts
**Family belief systems**		
**1. Make meaning of adversity**	**10**	
1.1 Manageability	6	Since then I’ve phoned the healthy parent once, he seemed very composed and indicated that he and his children are handling it reasonably well (ID 192, p. 5)
1.2 Normalization	2	Despite the mother’s poor prognosis, the family is doing well and the disease no longer takes over their lives to such an extent (ID 211, p. 14) (ID 211, p. 14)
1.3 Insights through illness	2	She is very focused on her recovery and is becoming more aware of things, which she has really learned to appreciate (ID 289, p. 7)
**2. Positive outlook**	**129**	
2.1 Confidence	10	The sick parent is getting better and better and feeling confident about her rehabilitation and professional reintegration (ID 161, p. 12)
2.2 Psychological well-being	24	The healthy parent and his children are doing well (ID 283, p. 14)
2.3 Psychological stress	64	The family seems to be under a lot of stress (ID 315, p. 1)
2.4 Courage and perseverance	5	The healthy parent seems strong and very clear, wishing his wife not to lose heart and still have some hope (ID 192, p. 1)
2.5 Self-care	2	She can now feel some calm returning to her life and is happy that the children are going to kindergarten and school again. She […] tries to take good care of herself (ID 309, p. 14)
2.6 Acceptance	8	In the first meeting with the sick parent, I have the feeling that there is a great deal of acceptance and clarity in the family and that they communicate well together (ID 310, p. 4)
2.7 Denial	10	I see the sick parent more as repressing some of the changes related to her diagnosis (ID 209, p. 4)
2.8 Ambivalence	8	Time and again, the family is very ambivalent about accepting the illness and the associated constraints, event not wanting to admit it and turning a blind eye in some instances (ID 193, p. 16)
**3. Transcendence and spirituality**	**4**	The family is in a good position, helped by their strong Catholic beliefs, and receive good support from the community […] (ID 175, p. 1)
**Family organization patterns**		
**4. Flexibility**	**102**	
4.1 Restructuring and adaptation	55	The family is handling the situation well. They are learning to adapt to and cope with the new structures and changes in their everyday lives (ID 192, p. 13)
4.2 Rigidity	6	The areas of responsibility of individual family member and what is assigned to each of them, including the distribution of tasks in the family, are very rigid and have developed over many years (ID 228, p. 15)
4.3 Being informed and seeking information	39	The family is well informed about support programs (ID289, S. 1)
4.4 Not being informed	2	Overall, I get the impression from the couple that they are not that well informed about the illness and/or that this is not really clear from the medical side either (ID 193, p. 2)
**5. Connectedness**	**122**	
5.1 Cohesion	30	Her family is still an important support and there is a good sense of togetherness (ID 161, p. 11)
5.2 Mutual support	53	The eldest son is currently unemployed and takes on a lot of chores at home (ID 248, p. 2)
5.3 Little cohesion and/or support	18	Family life seems to be managed by the sick parent alone. Overall, she also seems to be left alone with the challenging children, though appears at the same time reluctant/anxious to ask for more support from her husband (ID 301, p. 7)
5.4 Parents separated and/or contact difficulties	28	The sick parent is separated/divorced from her husband, and contact between the parents is very difficult, which is affecting the 13-year-old daughter (ID 326, p. 7)
**6. Social resources**	**208**	
6.1 Family members	82	When the mother is at the hospital for chemo, the mother’s cousin comes and takes care of the children (ID 153, p. 1)
6.2 Friends and acquaintances	27	There is a good social network in the neighborhood and among friends who take care of the children (ID 321, p. 7)
6.3 Professional support systems	81	The healthy parent reports that they received good psycho-oncological support from the clinic; even after the death of his wife there were further conversations there together with his son (ID 302, p. 7)
6.4 No social resources	28	There is no family support locally, grandma lives in “STATE,” the sister in “STATE”. The family seems quite alone (ID 178, p. 1)
**7. Economic resources**	**122**	
7.1 Employment	87	She receives sick pay, her husband goes to work as normal (ID 161, p. 6)
7.2 Unemployment	6	ET had himself laid off last year after the diagnosis, with the family now receiving Hartz IV support (ID 211, p. 1)
7.3 Well-off	18	The healthy parent is on sick leave, receiving sick pay, no financial worries (ID 331, p. 1)
7.4 Precarious financial situation	5	The sick parent lives on disability pension and rent-free in the mother’s inherited house. There are almost no reserves (ID 141, p. 2)
7.5 Financial support	11	The sick parent receives financial support from her brother where necessary (ID 315, p. 9)
**Family communication and problem-solving**		
**8. Clarity**	**51**	
8.1 Open communication	35	The children are well informed about their mother’s illness (ID 266, p. 4)
8.2 No open communication	17	The son is 10 years old and knows nothing about the disease (ID 294, p. 1)
**9. Open emotional expression**	**26**	
9.1 Sharing emotions	6	The father says he has good contact with his children and they are mourning together (ID 175, p. 1)
9.2 Acceptance of emotions and/or tolerance	7	In the first meeting with the sick parent, I have the feeling that there is a great deal of acceptance and clarity in the family and that they communicate well together (ID 310, p. 4)
9.3 No emotional sharing	14	In the conversation with the parents, it becomes clear that the two of them do not really communicate with each other much at an emotional level and also little as a family (ID 334, p. 7)
**10. Collaborative problem-solving**	**45**	
10.1 Working out solutions together	7	The parents are considering seeking couple therapy (ID 241, p. 11)
10.2 Taking chances/asking for help	8	The family wants prompt support to tell the “CHILD” that father has cancer. It is agreed that on the day of discharge I will go to their home to discuss the situation with the family (ID 144, p. 2)
10.3 Couple problems and/or no joint conflict resolution	31	The couple is in the process of separating. They argue with each other a lot (ID 312, p. 7)

Main categories (all deductive) have been highlighted in bold. Numbers per category correspond to the number of coded segments for that category.

### Qualitative analysis: dimensions and combinations of promoting family resilience processes

**Figure 1:**
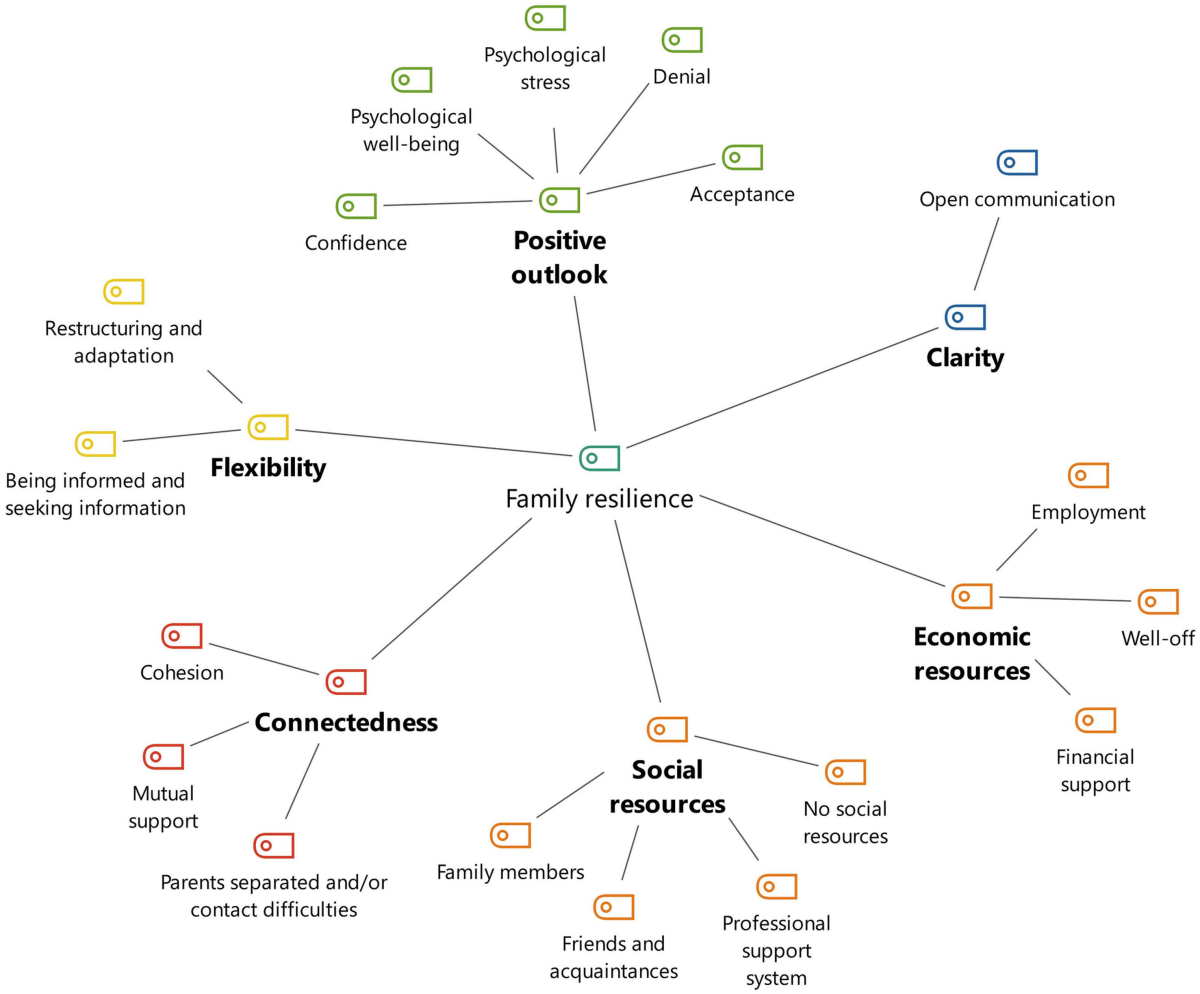
Concept map of the (combination of) family resilience categories that theoretically are resilience-promoting (according to Walsh’s model).

In the following, frequently coded combinations of main and subcategories of family resilience are described. Figure 1 shows a concept map of the frequently mentioned categories in the families. The main category with the most mentions was social resources (44 codings), followed by issues related to flexibility (24 codings) and economic resources (21 codings). Some text passages were also identified on positive outlook (14 codings) and family connectedness (13 codings).

#### Social resources

The Family-Scouts observed well-functioning social networks as well as experiencing support from family members (grandparents, aunts, siblings), friends or acquaintances, and professional help systems (Family-Scouts, home help, psycho-oncologists, psycho-therapists). The family members especially helped with child care and household tasks (e.g., shopping, cleaning).

#### Flexibility

The Family-Scouts observed adaptation to new life circumstances and flexible processes of restructuring everyday life. This included reducing work hours, restructuring daily routines, and also organizational flexibility (e.g., applying for rehabilitation, creating a living will and healthcare proxy, applying for sick pay). Families were also well informed about support services, treatment, and organizational matters or actively got in contact with the Family-Scouts to seek information on these topics. The children searched for information about parental cancer disease.

#### Employment

At least one parent either worked full-time, received sick pay, or was on parental leave. There were no reports of unemployment or financial problems.

#### Open communication

The analysis made it clear that there was open communication within the families. The illness or death of a parent was discussed openly, and partners and children were well informed. No concealment or withholding of information was observed.

## Discussion

The aim of the study was to qualitatively investigate resilience in families with parental cancer and minor children as well as family resilience categories that are resilience-promoting. Therefore, deductive [Family Resilience Framework (Walsh, 2003)] and inductive structuring qualitative content analysis (Kuckartz and Rädiker, 2020, 2022) of the Family-Scouts documentation (Dohmen et al., 2021) of supported families was performed. We could identify specific resilience processes described to different extents present within the families. Promoting family resilience processes were identified as a combination of social resources, flexibility, economic resources, and open communication.

### Family resilience in families with parental cancer and minor children

Overall, the findings regarding social resources, family cohesion, mutual support, flexibility, open communication, and psychological well-being as potential mechanisms for promoting resilience are consistent with existing assumptions about family resilience (Walsh, 2003; Visser et al., 2004; Black and Lobo, 2008; Choi and Yoo, 2015; Martin et al., 2015; Howard Sharp et al., 2017; Carr and Kellas, 2018). Open communication and psychological well-being that occurred in families in this study was also associated with resilience among family members in the cancer context (Carr and Kellas, 2018; Bajjani-Gebara et al., 2019).

Only few hints on transcendence and spirituality were identified in the Family-Scout documentation forms. This is in contrast to the results of previous research as spirituality was often attributed a central role in the resilience of a family (Black and Lobo, 2008; Martin et al., 2015). However, as spirituality or religiosity is assumed to interact highly with other resilience processes such as social resources (Bengel and Lyssenko, 2012), this could underlie the frequently confirmed positive relationship between spirituality and family resilience. The results of this study showed that spirituality was related to receiving social support, e.g., from the religious community. Furthermore, previous research attributed a key role in family resilience to collaborative problem-solving (Black and Lobo, 2008). In our sample, couple issues without collaborative problem-solving were mentioned frequently, even in families who otherwise showed many resilient processes. This may be due to the special situation of a family with one parent affected by cancer and minor children to care for, where role changes in different areas of life are especially difficult to manage for both parents (Hiltrop et al., 2021). Indeed, literature describing collaborative problem-solving as a core process of resilience has not focused on families affected by cancer (Black and Lobo, 2008).

Extensions of the specific context of this study emerged regarding the importance of economic resources and information-seeking. Literature has shown that satisfaction with the individual economic status contributed to the well-being of family members (McCubbin and McCubbin, 1988). A family’s economic resources may be used up in the course of parental cancer disease (Walsh, 2003) if the parent with primary income is no longer able to work due to the illness or if sick pay is insufficient to provide financial security for the family. Therefore, economic resources are an important consideration in the context of parental cancer with minor children (Phillips et al., 2021). Furthermore, information-seeking has also been found to promote resilience (Rutten et al., 2005). In the context of cancer, seeking disease-related information presumably has a more significant role than in other family crises (Walsh, 2003). This represents a possible explanation for the relevance of information-seeking found in this study through many inductive codings.

### Dimensions and combinations of promoting family resilience processes in families with parental cancer and minor children

Families received social support from family members, friends, and professional support systems. This is consistent with findings in previous literature on the resilience of individuals (Bengel and Lyssenko, 2012) as well as families (Patterson, 2002; Black and Lobo, 2008). Resilient families are able to admit when they need help and turn to their social environment or professional support systems to get it (Walsh, 1998). Flexibility is also considered a widely researched core process of (family) resilience (Walsh, 2003; Black and Lobo, 2008; Martin et al., 2015). This is consistent with findings from this study. Families demonstrated flexible processes of restructuring to changing life circumstances with the cancer disease. Active seeking of information on topics such as diagnosis, treatment, possible support services, and social-legal issues was also observed. Previous literature on this topic has shown that parents of children with cancer demonstrated that they used information-seeking as a problem-focused coping strategy (Rutten et al., 2005). An important aspect of resilience among patients with chronic pain was characterized by a sense of control, which sometimes developed from information-seeking (Rolbiecki et al., 2017). Furthermore, economic security with income in the form of salary, sick pay, or parental benefits was coded as promoting family resilience. Resilience research in the family context has only addressed families’ economic resources to a limited extent, focusing here on the absence rather than the presence of this resource (Conger et al., 1990; Fogel, 2017). The findings of this study argue for not neglecting the importance of economic resources to the family resilience of families with parental cancer and minor children. The role of open communication within families which has been recognized in literature (Choi and Yoo, 2015; Carr and Kellas, 2018) was supported in the present research. Past research showed that open communication in families was characterized by directness, clarity, and honesty (Lindsey and Mize, 2001). In the face of parental cancer, clarification of information about topics such as diagnosis, treatment, side effects, and prognosis is particularly important, as a lack of open communication may cause children to fill existing gaps in knowledge about these topics with anxiety or even feeling guilty (Garmezy et al., 1984; Kühne et al., 2012; Faccio et al., 2018).

### Limitations and strengths

In this study, the relevance of the corresponding resilience processes was derived from the number of mentions in the documentation forms of the Family-Scouts (coded segments). It should be noted that some processes can be more easily observed by a person from outside the family (e.g., social and economic resources) and others are more difficult to observe (open expression of emotions within the family, spirituality). The Family-Scouts commented on difficulties in assessing such issues in their documentation forms. Furthermore, processes such as transcendence and spirituality are not consistently defined in the literature (Walach, 2022). Therefore, it can be assumed that the number of mentions in the documentation forms does not automatically indicate the actual relevance of the corresponding resilience processes. One important example of this limitation might be the category “religion and spirituality”: religion and spirituality tend to be private matters in Germany and are rarely addressed in everyday discourse and patient care. This topic could be better explored with qualitative interviews than with routine documentations by the Family-Scouts (social workers).

Social support was possibly asked about more actively, so that only at this point, for example, an involvement in a religious community became clear. Termination of contact with the Family-Scouts or withdrawal of consent to the study resulted in a reduced number of completed documentation forms in some families. However, in order to increase the validity of the qualitative content analysis conducted, the data were analyzed by two researchers with different professional backgrounds (psychology and sociology), and in case of discrepancies an agreement was reached before data analysis. Furthermore, the data are based on documentation within one cancer center in Germany. The generalizability of the results is limited to families with an oncological disease who were treated at this university hospital in Germany. Nevertheless, data on family resilience of families with parental cancer and minor children is very rare, and here, unique data from a new intervention (“F-SCOUT” study) could be qualitatively analyzed with common qualitative methods.

### Implications for research and practice

The study showed that Walsh’s framework of family resilience (Walsh, 2003) can be applied to families with parental cancer and minor children and contributes to an improved understanding of how resilience can emerge and be supported in families. The resilience process, i.e., finding a way to adapt positively to the new situation created by the cancer disease (Luthar et al., 2000), and being able to move consciously forward as a family in this situation (Southwick et al., 2014), was connected to social and economic resources, flexibility, and open communication. As changes in resilience could not be analyzed over the three measurement points, future studies can address this research gap. The results also underline the importance of information-seeking as an important process of coping with cancer disease (Rutten et al., 2005). Long-term mental health outcomes might be affected as (family) resilience has been associated with higher subjective well-being (Bajjani-Gebara et al., 2019), stress coping abilities (Patterson, 2002; Connor and Davidson, 2003), and higher quality of life (Kurz et al., 2014; Molina et al., 2014; Li et al., 2016) of cancer patients and their relatives. The findings can also serve as inspiration for future research by providing starting points for the formulation of quantitative hypotheses, e.g., concerning health outcomes for individual family members or on family level. They may provide a basis for the development of a questionnaire assessing resilience in families with parental cancer and minor children. They may also help to adapt previous questionnaires for assessing family resilience (Walsh, 2003; Zhou et al., 2020).

The results provide practical implications for how healthcare providers (HCP) can support families with parental cancer and minor children. HCP should be sensitized for families with parental cancer and minor children in general. Furthermore, as information-seeking is a possible resilience-building process, this is an opportunity for HCP to effectively support families by providing disease-related (evidence-based) information. A focus on families with less developed resilience processes can help identify families at risk early and offer them increased support. Also, potential consequences such as the impact on minor children (Flechtner et al., 2011; Heinemann and Simeit, 2017) could be more specifically regulated in prevention programs as resilience-based interventional studies show positive results in reducing psychopathology in children (Garmezy et al., 1984; Fritz et al., 2018). Within the German healthcare system, the interprofessional care of affected families seems important as resilience has emerged as an important element of psychological care for cancer patients (Molina et al., 2014). Examples include social support from healthcare workers (e.g., Family-Scouts), interventions to promote restructuring and adjustment, and providing information about the disease, its treatment, and possible support services. Family discussions to promote open communication could also help affected families develop an adaptive approach to the disease and its associated challenges (Zomerlei, 2015; Lundquist, 2017). Financially less well-off family systems with a parent with cancer could be strengthened by access to governmental financial assistance. These findings provide insights and support for intervention and prevention approaches to strengthen family resilience in families with parental cancer and minor children.

## Conclusion

The study indicates that family resilience processes describe and analyze challenges of families with parental cancer and minor children in a comprehensive and multidimensional way. For HCP this might help to better understand the functioning of families entrusted to their care. For researchers this might help to provide insights and support for intervention and prevention approaches to strengthen family resilience in families with parental cancer and minor children. The findings are consistent with existing assumptions about family resilience (Walsh, 2003; Choi and Yoo, 2015) in terms of the importance of social resources, family cohesion, mutual support, flexibility, open communication, and psychological well-being. In contrast to the results of previous research (Black and Lobo, 2008; Martin et al., 2015), the findings on spirituality and collaborative problem-solving in this study indicate less importance. In turn, the findings on economic resources and information-seeking provide a valuable extension to the literature in the context of families with parental cancer and minor children. Further research on resilience in these families is needed especially to provide guidance for integrating findings into clinical practice (Veach and Nicholas, 1998; Visser et al., 2004; van Schoors et al., 2015).

## Data availability statement

The raw data supporting the conclusions of this article will be made available by the authors on reasonable request, without undue reservation.

## Ethics statement

The studies involving humans were approved by the Ethics Committee of the Medical Faculties of the RWTH Aachen University (EK195/18), University of Bonn (267/18) and the Heinrich-Heine University Düsseldorf (2018-215). The studies were conducted in accordance with the local legislation and institutional requirements. Written informed consent for participation in this study was provided by all participants themselves or the participants’ legal guardians/next of kin.

## Author contributions

AP-M, NE, TB, FG, AI, and AK contributed to the conception and design of the study. RB, AP-M, CH, LH, HN, AK, and FG organized the database. CH and JS performed the data analysis. CH, JS, LH, NE, HN, AP-M, RB, and FG interpreted the results. CH, JS, and FG wrote the first draft of the manuscript. All authors commented and revised the manuscript critically and contributed to the manuscript revision, read, and approved the submitted version.
