# Supplementary material for: Family resilience of families with parental cancer and minor children: a qualitative analysis
Source: Front Psychol. 2024 Jan 19;14:1251049. doi: 10.3389/fpsyg.2023.1251049 (PMC10836593; doi:10.3389/fpsyg.2023.1251049)
Supplement: Supplementary file 1 [file Table_1.docx]

| **Situation of the family in relation to the disease** | | |
| --- | --- | --- |
| - Who is ill and at what stage? - Situation of the healthy parent (living at home/able to work...?) - How many children and how old? - Which treatment is currently pending? - How well informed is the family about help (in terms of whether counseling might be sufficient)? - Does everyone know about the disease? | | |
| **Special features of the family** | | |
|  | | |
| **Organization** | **Actual state** | **Demand** |
|  | | |
| Household management |  |  |
| Supervision of |  |  |
| Child 1 |  |  |
| Child 2 |  |  |
| Child 3 |  |  |
| Support for the sick parent |  |  |

| **Organization** | **Actual state** | **Demand** |
| --- | --- | --- |
|  | | |
| Sickness benefit | Select an element. |  |
| Transitional allowance | Select an element. |  |
| ALG I | Select an element. |  |
| Social security | Select an element. |  |
| Severely disabled person's pass | Select an element. |  |
| Rehabilitation measure | Select an element. |  |
| Follow-up treatment | Select an element. |  |
| Reduced earning capacity pension | Select an element. |  |
| Housing benefit/encumbrance allowance | Select an element. |  |
| Child benefit supplement | Select an element. |  |
| Education and participation package | Select an element. |  |
| Hardening fund | Select an element. |  |
| Relief fund of the Federal President | Select an element. |  |
|  | Select an element. |  |
|  | Select an element. |  |

| **Work orders to be derived/plan of action** | | | | | |
| --- | --- | --- | --- | --- | --- |
| 1 |  | | | | |
| 2 |  | | | | |
| 3 |  | | | | |
|  |  | | | | |
|  |  | | | | |
| **Emotional coping with illness** | | | | | |
|  | | | **Displacement** | **Acceptance** | **Load** |
|  | | | | | |
| Family | | |  |  | Wählen Sie ein Element aus. |
|  | | |  |  |  |
| Mother | | ill | ☐ | ☐ | Wählen Sie ein Element aus. |
| Father | | healthy | ☐ | ☐ | Wählen Sie ein Element aus. |
| Child 1 | | | ☐ | ☐ | Select an element. |
| Child 2 | | | ☐ | ☐ | Select an element. |
| Child 3 | | | ☐ | ☐ | Select an element. |
|  | | | ☐ | ☐ | Select an element. |
|  | | | ☐ | ☐ | Select an element. |

| Need further support? |  | Yes |  | no |  |
| --- | --- | --- | --- | --- | --- |
|  | | | | | |
|  |  | Family SCOUT |  | Other: |  |
|  | | | | | |

| **Work orders to be derived/plan of action** | |
| --- | --- |
|  |  |
|  |  |
|  |  |
|  |  |
|  |  |

| **Stimulating open communication within the family** | | | | | |
| --- | --- | --- | --- | --- | --- |
|  | | | | | |
| **Assessment of the family's communication** | | | | | |
|  | | | | | |
|  | Not open at all | Barely open | Mediocre | Fairly open | Very open |
|  | | | | | |
| Between the partners |  |  |  |  |  |
|  | | | | | |
| With the children |  |  |  |  |  |
|  | | | | | |
| With outsiders |  |  |  |  |  |

| **Work orders to be derived/plan of action** | |
| --- | --- |
|  |  |
|  |  |
|  |  |
|  |  |
